# SENP1 promotes MCL pathogenesis through regulating JAK-STAT5 pathway and SOCS2 expression

**DOI:** 10.1038/s41420-021-00578-x

**Published:** 2021-07-26

**Authors:** Yali Zhang, Yanni Ma, Guixian Wu, Mingling Xie, Chengxin Luo, Xiangtao Huang, Feng Tian, Jieping Chen, Xi Li

**Affiliations:** 1 Department of Hematology, Southwest Hospital, Army Medical University (Third Military Medical University), Chongqing, China; 2 Department of Hepatobiliary Surgery, Southwest Hospital, Army Medical University (Third Military Medical University), Chongqing, China; 3 Institute of Infectious Diseases, Southwest Hospital, Army Medical University (Third Military Medical University), Chongqing, China

**Keywords:** Lymphoma, Glycobiology

## Abstract

Mantle cell lymphoma (MCL) is highly aggressive and its treatment remains challenging, understanding its pathogenesis is critical for future targeted therapy. SUMO specific proteases 1 (SENP1) is an important protein that regulates the balance between SUMOylation and deSUMOylation. We found that SENP1 was upregulated in MCL patient samples and cell lines. Knockdown of SENP1 could inhibit the proliferation and promote the apoptosis of MCL cells. We also found that SENP1 knockdown caused inhibition of the JAK-STAT5 pathway and upregulation of tumor suppressor cytokine signaling 2 (SOCS2). Moreover, MCL tumor growth in vivo was significantly suppressed after SENP1 knockdown in a xenograft nude mouse model. In summary, our results showed that SENP1 is involved in the pathogenesis of MCL and may be a potential therapeutic target.

## Introduction

Mantle-cell lymphoma (MCL) is a highly malignant and aggressive B-cell non-Hodgkin’s lymphoma (NHL) which accounts for 5–10% of all NHL [1]. The disease is characterized by cyclin D1 overexpression caused by chromosomal translocation t (11;14) (q13; q32) [2, 3]. Although the treatment of MCL patients has achieved significant advances in chemotherapy, targeted therapy and immunotherapy, it remains challenging since most of MCL patients still experience early and frequent relapse and finally die from the disease. New therapeutic targets are urgently needed to be developed for these patients.

SUMOylation is a crucial post-translational modification characterized by covalent conjugation of small ubiquitin-like modifiers (SUMOs) to target proteins [4, 5], which exerts indispensable roles in various cellular processes, such as protein localization, gene transcription, and DNA replication [6–8]. The modification is a dynamic reversible process and the deconjugation of SUMOylation (deSUMOylation) is mediated by SUMO-specific proteases (SENPs) [9, 10]. Among the seven members of SENPs, SENP1 is wildly distributed in the nucleus and also expressed in cytoplasm with functions of deconjugating a great number of SUMOylated proteins and processing the precursor SUMO to generate mature SUMO, which plays the most potent regulatory role in maintaining the balance of SUMOylation and deSUMOylation [11–13]. The dysregulated expression of SENP1 has been implicated in the pathogenesis of various cancers, including colon cancer [14], prostate cancer [15, 16], etc. Moreover, SENP1 specific inhibitors have shown promising therapeutic effects in a variety of cancers, which suggested that SENP1 may be an effective potential therapeutic target in the treatment of cancers. However, little is known about the role of SENP1 in MCL.

In this study, we investigated the function and the underlying molecular mechanism of SENP1 in MCL pathogenesis. We found SENP1 was upregulated in MCL patient samples and cell lines; knockdown of SENP1 inhibited MCL cell proliferation and promoted cell apoptosis, and also inhibited MCL tumor growth in mice; mechanically, knockdown of SENP1 resulted in an increased expression of tumor suppressor cytokine signaling 2 (SOCS2) by inhibiting the JAK-STAT5 activity. These findings show that SENP1 is involved in the pathogenesis of MCL and may be a potential therapeutic target.

## Materials and methods

### Chemicals and reagents

Cell culture reagents (RPMI 1640 medium, DMEM medium and fetal bovine serum) were provided by Gibco Co. (Grand Island, NY). All primers were provided by Sangon Biotech, and viral constructs were provided by Genechem Co. (Shanghai, China). Trizol RNA extraction kit was provided by Invitrogen Co., PrimeScript RT reagent Kit and SYBR Green real time quantitative PCR kit were provided by TaKaRa. NE-PER™ Nuclear and Cytoplasmic Extraction Reagents were purchased from Thermo Fisher (Shanghai, China). Click-iT® EdU Imaging Kit (#C10425) was from Invitrogen (Carlsbad, CA). In-Situ Cell Death Detection Kit, TMR red (#12156792910) was from Roche (Basel, Switzerland). APC Annexin V Apoptosis Detection Kit with PI (#640932) was purchased from BioLegend (San Diego, CA, USA). The antibodies against SENP1 (#ab108981) and GAPDH (#ab20272) were purchased from Abcam (Cambridge, England). Antibodies for SOCS2 (#2779), Rb (#9313), p-Rb (#8180), cyclinD1 (#4874), β-actin (#2062), Bid (#8762 S), Caspase 9 (#9508 T), C-caspase 3 (#9661 S), PARP (#5625 T), p53 (#9282), STAT5 (#94205), p-STAT5 (#4322 and #9359), LaminB1 (#13435), Ki67 (#12202) and DAPI (#4083) were obtained from Cell Signaling Technology (Shanghai, China). Recombinant human erythropoietin (EPO) injection (EPIAO, #S19980073) was purchased from Shenyang Sansheng Pharmaceutical Co. (Shenyang, China).

### Patient specimens

Lymph node specimens were obtained from 4 patients with previously untreated MCL and 4 patients with reactive lymph node enlargement who underwent lymph node biopsy at the Southwest Hospital affiliated to Amy Medical University (Third Military Medical University). The specimens were fixed in 10% formalin and processed for paraffin embedding. Informed consent was received from each patient, and the ethics committee of the Southwest Hospital approved the study.

### Immunohistochemical staining

Immunohistochemistry was performed to detect the expression of SENP1, Ki67, and p-STAT5. The detailed process has been described in our previous article [17]. In brief, the formalin-fixed, paraffin-embedded specimens were sliced into 4 µm tissue sections, then depolished with xylene and rehydrated with an alcohol solution. After antigen retrieval, the tissue sections were incubated with anti-SENP1, anti-Ki67, or anti-p-STAT5 primary antibody for 24 hours (h) at 4 °C, and then washed with PBS and incubated with peroxidase-conjugated goat anti-human/mouse secondary antibody (1:200 dilution, #ab6858 and #ab150113, Abcam, USA) for 1 h at room temperature. Five fields were randomly selected from each section under a light microscope (100×, BX51, Olympus, Japan) fitted with a digital camera (DP72, Olympus, Japan). Image-Pro Plus 6.0 software was used to quantitively analyze the images. Quantification of SENP1, Ki67, and p-STAT5 was determined by mean of integrated optical density (IOD).

### GEO datasets analysis

Normalized microarray data in the Gene Expression Omnibus (GEO) database (www.ncbi.nlm.nih.gov/geo/) under accession number GSE32018 was downloaded and analyzed, including 24 freshly frozen lymph nodes from MCL patients and 13 freshly frozen normal lymph node or tonsil tissues as controls. The probe-level data in CEL files were converted into expression profiles in R software (https://www.r-project.org/) and the robust multiarray average (RMA) algorithm with affy package was used to correct and normalize expression profiles data. Expression levels of SENP1 and SOCS2 were compared between MCL samples and control samples, and their correlation was analyzed in all samples.

### Cell lines and cell culture

Human MCL cell lines (MAVER-1, REC-1, Mino, Jeko-1) were purchased from ATCC (Beijing, China) and human normal B cell line (Wil2-s) from Institute of Cell Resource Conservation of China National Institute for Food and Drug Administration (Beijing, China), respectively in 2018. They were authenticated by the suppliers and tested by PCR to be free of Mycoplasma contamination in November 2018 upon completion of the experiments. The cells were cultured in RPMI 1640 medium, supplemented with 10% fetal bovine serum and 1% penicillin-streptomycin (Gibco) at 37 °C in 5% CO_2_ incubator.

### RT-qPCR

Total RNA of cells was extracted with Trizol kit (TaKaRa, Shiga, Japan). A total of 1ug RNA was reverse-transcribed to synthesis complementary DNA (cDNA) using PrimeScript RT reagent Kit (TaKaRa). The cDNAs were quantified by quantitative PCR (qPCR) with SYBR Green real time quantitative PCR kit (TaKaRa). β-actin was used as an internal control. The primers used in the study were shown in Table S1. Each sample was analyzed in triplicate. Relative expression levels from three independent experiments were calculated with the 2^-△△CT^ method.

### Western blot

Cellular samples were lysed with RIPA buffer containing a protease inhibitor cocktail as previously described. Nuclear and cytoplasmic proteins were extracted using the NE-PER™ Nuclear and Cytoplasmic Extraction Reagents (Thermo Fisher, Shanghai, China) according to manufacturer’s protocol. The protein concentration was determined using Enhanced BCA Protein Assay Reagent (Beyotime, Jiangsu, China). Equal quantities of proteins from each sample were separated by 10% SDS-PAGE, and then transferred to PVDF membranes (Bio-Rad, 162–0177). After blocking with 5% fat-free dry milk, the membranes were incubated with specific primary antibodies overnight at 4 °C and corresponding HRP-conjugated secondary antibodies for 2 h at room temperature. Protein bands were visualized with an enhanced chemiluminescence system (Perkin-Elmer Life Sciences, Boston, USA). Band intensities were quantified using ImageJ software, and β-actin, LaminB1 or GAPDH was used as internal reference.

### Construction of stable SENP1 knockdown cell lines

Lentiviral vectors encoding three different short hairpin RNAs (shRNAs), targeting human SENP1 (named as shSENP1-1, shSENP1-2 and shSENP1-3 respectively, listed in Table S1), were purchased from Shanghai Genechem of China (Shanghai, China).

MCL cells (MAVER-1, REC-1, Mino, Jeko-1) were transfected with the shSENP1 lentivirus or empty lentiviral vector (named as shCon) with polybrene (8 μg/ml, Sigma-Aldrich, Missouri, USA). The transfected cells were then subjected to selection by puromycin (3.0 μg/ml) for 4–5 days. Knockdown of SENP1 in the stable cells was verified by RT-qPCR and western blot.

### Cell proliferation assay

The cell proliferation was measured with Cell Counting Kit-8 (CCK-8) assay according to manufacturer’s direction (Beyotime, Shanghai, China). In detail, MCL cells were seeded in 96-well plates at a density of 5000 cells/well and cultured in RPMI 1640 with 10% FBS at 37 °C in 5% CO_2_ incubator for 5 days. The optical density (OD) was measured at 450 nm on each day. The cell viability of the Jeko-1 and Mino cells transfected with shSENP1 was normalized with the value from the cells transfected with shCon. Each experiment was repeated in triplicate.

### Cell cycle analysis

MCL cells were seeded into 6-well plates (1×10^4^ cells per well), 10 mM Edu (Invitrogen) was added to each well and incubated at 37 °C for 2 h. Edu staining were performed with the Click-iT Edu kit (Invitrogen) according to manufacturer’s protocol. Before being analyzed on FACScan flow cytometer (BD Biosciences, San Jose, CA, USA), the cells were stained with DAPI at room temperature for 5 minutes (min). The cell cycle distribution was calculated using the FlowJo software (Treestar, Ashland, OR, USA). Cells were divided into G1 or G2 phase according to the DNA content displayed by DAPI. Each experiment was repeated in triplicate.

### Cell apoptosis assay

Cell apoptosis was evaluated by flow cytometric analysis with Annexin V-APC and propidium iodide (PI) double staining according to the manufacturer’s instructions (BD Biosciences). In brief, 1 × 10^6^ cells were stained with 2 ul of Annexin V-APC and 5 μl of PI (50 μg/ml) in 1× binding buffer (10 mM of HEPES, pH 7.4, 140 mM of NaOH, and 2.5 mM of CaCl_2_) for 15 min in the dark at room temperature. Quantification of apoptotic cells was analyzed by FACScan flow cytometer and calculated using the FlowJo software. Cells stained positive for Annexin V-APC, PI, or both were considered apoptotic cells. Each experiment was repeated in triplicate.

### Cells treated with EPO

Jeko-1 and Mino cells transfected with shSENP1-3 were treated with EPO at a concentration of 50 IU/ml and 40 IU/ml, respectively. After the cells treated with EPO for 1 days, SOCS2 mRNA level was detected by RT-qPCR, and protein levels of SOCS2, p-STAT5 and STAT5 were detected by western blot. Meanwhile, cell proliferation was measured with CCK-8 assay on day 0, 1, 2, and 3, cell apoptosis was evaluated by flow cytometric analysis with Annexin V-APC and PI double staining on day 2. Each experiment was repeated in triplicate.

### mRNA sequencing and functional enrichment analysis

mRNA sequencing (mRNA-seq) was performed to analyze differentially-expressed genes (DEGs) between Jeko-1 cells with shCon and shSENP1, which was constructed by LC Science, Hangzhou, China. The DESeq2 R package (1.26.0) was used to obtain DEGs between groups. When adjusted *P* < 0.05, DEGs were considered as significantly different. Genes with an absolute value of log2 (fold change) (log2 FC) > 1 were identified as significantly differentially expressed genes. Heatmap was generate by heatmap version 1.0.10.

Kyoto Encyclopedia of Genes and Genomes (KEGG) enrichment analyses were performed to reveal the significant pathways of DEGs using the clusterProfiler R (v3.13.0) package. KEGG terms with corrected *P* < 0.05 were considered significantly enriched by DEGs. Gene Set Enrichment Analysis (GSEA) was performed using the GSEA (v2.2.3, http://software.broadinstitute.org/gsea/downloads.jsp) with MSigDB-curated gene sets (c2.cp.kegg.v6.2.symbols.gmt).

### In vivo xenograft studies

Four-week-old male BALB/c nude mice were obtained from the Laboratory Animal Center of Army Medical University (Third Military Medical University). They were randomly assigned into two groups (shCon and shSENP1-3). To develop xenograft mouse model, 1.5 × 10^7^ stably transfected Jeko-1 cells with shCon or shSENP1-3 suspended in 100 μl serum-free medium plus 100 μl Matrigel, were subcutaneously injected to the right flanks of nude mice at the age of 6 weeks. Tumor volumes were measured and recorded every 2 days. The tumor volume was calculated using the equation reported in a previous study: V = (lengthlculat^2^)/2 (ref. [18]). Mice were finally killed at the 21st day after cell injection to obtain the tumor tissues. The expression of SENP1 in the tumor tissue was detected both by immunohistochemistry and western blot. Ki67 and p-STAT5 were also detected in the tumor tissue by immunohistochemistry. Each group contained 5 mice. All mouse experiments were performed without blinding and conducted in accordance with the Public Health Service Policy on Humane Care and Use of Laboratory Animals. The Experimental Animal Ethics Committee of Army Medical University approved the experiments.

### TUNEL assay

The harvested mouse tumor tissues above were fixed in 10% formalin and embedded with paraffin. The number of apoptotic cells in the tissues was detected using TUNEL assay (In-Situ Cell Death Detection Kit, Roche) according to the manufacturer’s protocol. In brief, after deparaffinized and rehydrated, 5-μm tissue sections were incubated with proteinase K, and the endogenous peroxidase activity was blocked with hydrogen peroxide. The sections were then incubated with the terminal TdT/ nucleotide mixture at 37 °C for 1 h. Next, the reaction was stopped and the sections were rinsed with phosphate-buffered saline (PBS). Nuclear labeling was developed with horseradish peroxidase and diaminobenzidine. Hematoxylin was used for counterstaining. Cells with brown nuclei were considered as apoptotic cells. The percentage of apoptotic cells was calculated by an investigator who was blinded to the group assignment.

### Statistical analysis

Data were expressed as mean ± standard deviation (SD). Statistical significance of differences between groups was determined using two-sided Student’s *t* test. The correlation between SENP1 and SOCS2 expression was analyzed by Pearson correlation. Sample sizes were determined according to previous literature. A *P* < 0.05 was considered as statistically significant. All statistical analyses were performed using SPSS 20.0 or GraphPad Prism 6.0 software.

## Results

### SENP1 is upregulated in mantle cell lymphoma

**Fig. 1:**
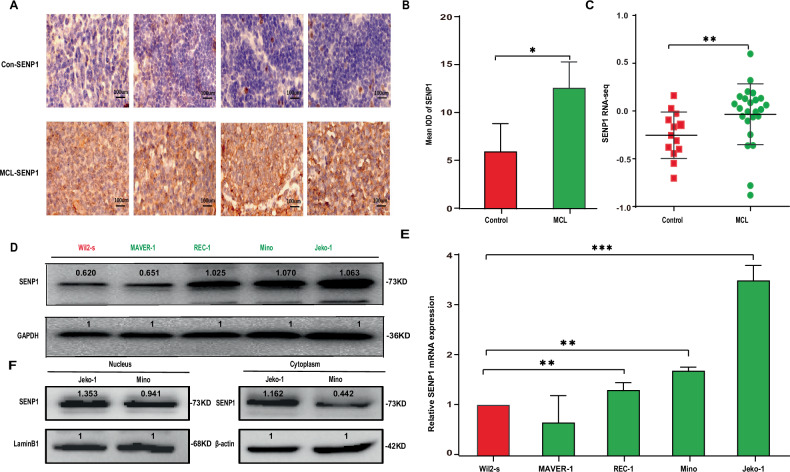
SENP1 is highly expressed in human MCL samples and cell lines. **A** Representative immunohistochemical images of SENP1 staining in human MCL samples and control samples (*n* = 4 per group). **B** The mean integrated optical density (IOD) of SENP1 protein in human MCL samples and control samples was calculated using Image-Pro Plus 6.0 software. **C** SENP1 mRNA level in human MCL samples and control samples was analyzed through extracting GEO RNA-seq data. SENP1 protein level was determined by western blot (**D**), SENP1 mRNA level was measured by RT-qPCR (**E**) in human normal B cell line (Wil2-s) and MCL cell lines (MAVER-1, REC-1, Mino and Jeko-1)**. F** Nuclear and cytoplasmic protein level of SENP1 was determined by western blot after subcellular fractionation in Jeko-1 and Mino cells. Data were presented as mean ± SD and analyzed by two-sided Student’s *t* test. *n* = 3; *P < 0.05, **P < 0.01, and ***P < 0.001.

To test the expression of SENP1 in MCL, we firstly detected the expression of SENP1 in human MCL specimens and reactive lymph node enlargement specimens by immunohistochemistry, which showed that the SENP1 protein expression in the MCL specimens was significantly higher than that in the reactive lymph node enlargement specimens (Fig. 1A, B). SENP1 mRNA expression was also analyzed in 24 freshly frozen tumor lymph nodes from MCL patients and 13 freshly frozen normal lymph nodes or tonsil tissues by extracting data from GEO database, and was confirmed to be significantly upregulated in MCL specimens compared with that in normal specimens (Fig. 1C). Furthermore, we detected the protein and mRNA expressions of SENP1 in several human MCL cell lines (MAVER-1, REC-1, Mino, Jeko-1) and human normal B cell line Wil2-s, which demonstrated that SENP1 mRNA and protein expressions were upregulated in REC-1, Mino, and Jeko-1 cells compared with Wil2-s cells (Fig. 1D, E). In addition, by measuring nuclear and cytoplasmic protein expression levels of SENP1 after subcellular fractionation in Jeko-1 and Mino cells, we found that SENP1 was distributed in both nucleus and cytoplasm, and it was more concentrated in the nucleus (Fig. 1F). These results confirm that SENP1 is upregulated in human MCL tissues and cells.

### SENP1 knockdown inhibits proliferation of MCL cells

**Fig. 2:**
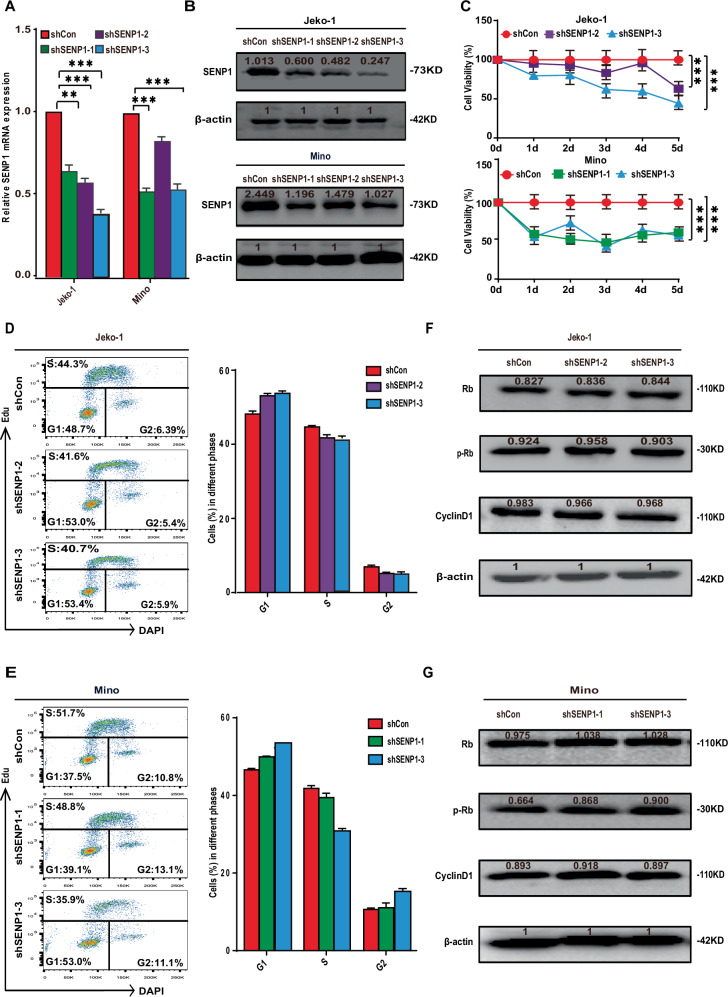
SENP1 knockdown inhibits proliferation of MCL cells. SENP1 mRNA level was measured by RT-qPCR (**A**), and SENP1 protein level was measured by western blot (**B**) in Jeko-1 and Mino cells after transfected with shSENP1 or shCon. **C** Cell viability was evaluated by CCK8 array in Jeko-1 and Mino cells after transfected with shSENP1 or shCon. The cell viability of the shSENP1 group was normalized with the value from the shCon group. The cell cycle distribution of the transfected Jeko-1 (**D**) and Mino (**E**) cells was assessed by Edu and DAPI staining and flow cytometric analysis. The proportion of cells in G1, S, and G2 phases was shown in the right histogram. The Rb, p-Rb and CyclinD1 protein levels were measured by western blot in the transfected Jeko-1 (**F**) and Mino (**G**) cells. Data were presented as mean ± SD and analyzed by two-sided Student’s *t* test. *n* = 3; **P < 0.01, and ***P < 0.001.

To clarify the influence of SENP1 on proliferation and cell cycle progression of MCL cells, we constructed stable Jeko-1 and Mino cells with SENP1 knockdown using three different lentiviral shRNAs targeting SENP1 (named as shSENP1-1, shSENP1-2 and shSENP1-3 respectively). Stable Jeko-1 or Mino cells transfected with empty lentiviral vector (named as shCon) were used as the negative control. RT-qPCR and western blotting demonstrated that stable Jeko-1 cells with shSENP1-2 or shSENP1-3 and stable Mino cells with shSENP1-1 or shSENP1-3 had more significantly decreased SENP1 mRNA and protein expression (Fig. 2A, B). The proliferation of the above stable cells was significantly inhibited as compared with that of control cells confirmed by the CCK-8 array (Fig. 2C). We also explored whether SENP1 knockdown can affect the cell cycle of MCL cells by flow cytometric analysis, and the results showed there was no significant difference in cell cycle distribution between stable cells with SENP1 knockdown and control cells (Fig. 2D, E), which was further demonstrated by detecting the expression of cell cycle related proteins, including Rb, p-Rb and CyclinD1 (Fig. 2F, G). Collectively, these results show that SENP1 knockdown inhibits MCL cell proliferation but without significant effect on cell cycle distribution.

### SENP1 knockdown increases apoptosis of MCL cells

**Fig. 3:**
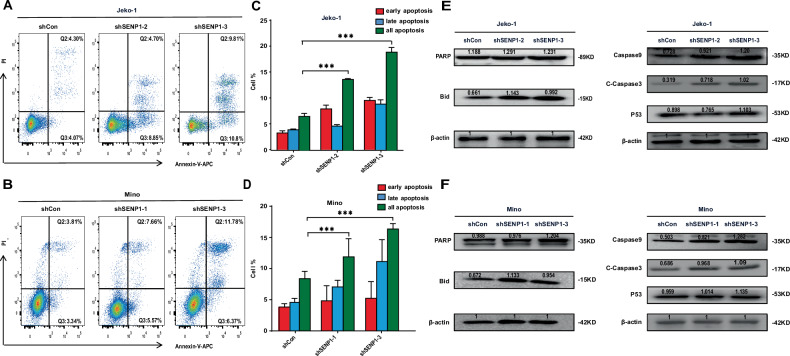
SENP1 knockdown increases apoptosis of MCL cells. The apoptosis of transfected Jeko-1 (**A**) and Mino (**B**) cells was measured by Annexin V-APC and PI double staining and flow cytometric analysis. Representative flow cytometry patterns from different groups were shown. The percentages of apoptotic cells of transfected Jeko-1 (**C**) and Mino (**D**) cells were calculated according to the flow cytometric results. The protein levels of PARP, Bid, Caspase9, C-Caspase3 and P53 were measured by western blot in transfected Jeko-1 (**E**) and Mino (**F**) cells. Data were presented as mean ± SD and analyzed by two-sided Student’s *t* test. *n* = 3; ***P < 0.001.

To test whether SENP1 knockdown can induce apoptosis in MCL cells, we detected the apoptosis of stable Jeko-1 cells with shSENP1-2 or shSENP1-3 and stable Mino cells with shSENP1-1 or shSENP1-3 by Annexin V-APC and PI double staining. The percentages of apoptotic cells were significantly higher in the Jeko-1 and Mino cells with SENP1 knockdown compared with the control cells (Fig. 3A–D). Correspondingly, SENP1 knockdown in Jeko-1 and Mino cells caused the activation of Caspase9 and C-caspase3, and the cleavage of Bid and PARP (Fig. 3E, F). These results indicate that SENP1 knockdown induces MCL cell apoptosis.

### SENP1 knockdown promotes SOCS2 expression by inhibiting JAK-STAT5 pathway in MCL cells

**Fig. 4:**
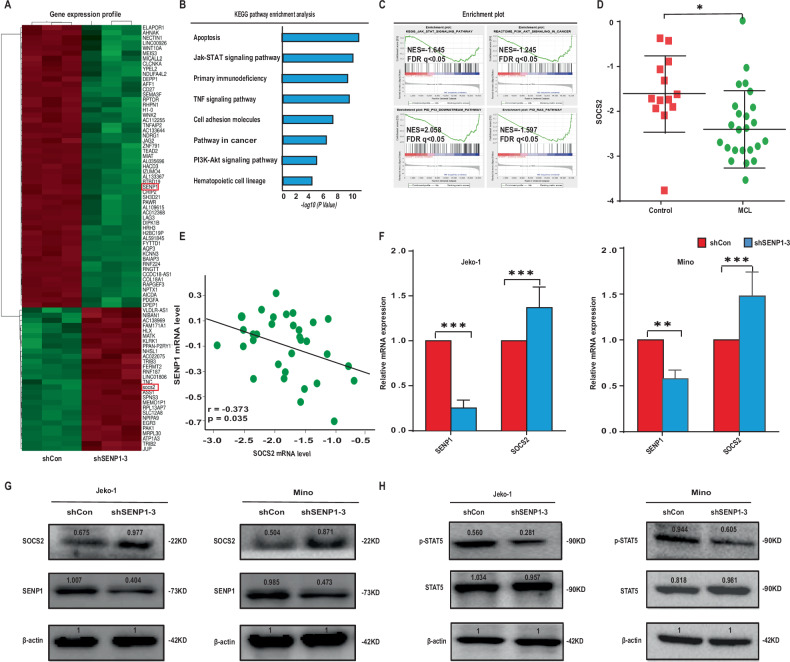
SENP1 affects JAK-STAT5 signaling pathway in MCL. **A** Heatmap revealed differentially expressed genes between Jeko-1 cells transfected with shCon and shSENP1-3. **B** KEGG enrichment analysis showed that differentially expressed genes were enriched at different signaling pathways after SENP1 knockdown. **C** GSEA was performed using differentially expressed genes. **D** SOCS2 mRNA level in human MCL samples and normal control samples was analyzed through extracting GEO RNA-seq data. **E** Pearson correlation analysis between SENP1 mRNA level and SOCS2 mRNA level using GEO RNA-seq data. SOCS2 mRNA level was measured by RT-qPCR (**F**), SOCS2 (**G**) and p-STAT5 (**H**) protein levels were measured by western blot in transfected Jeko-1 and Mino cells. Data were presented as mean ± SD and analyzed by two-sided Student’s *t* test. *n* = 3; *P < 0.05, and ***P < 0.001.

**Fig. 5:**
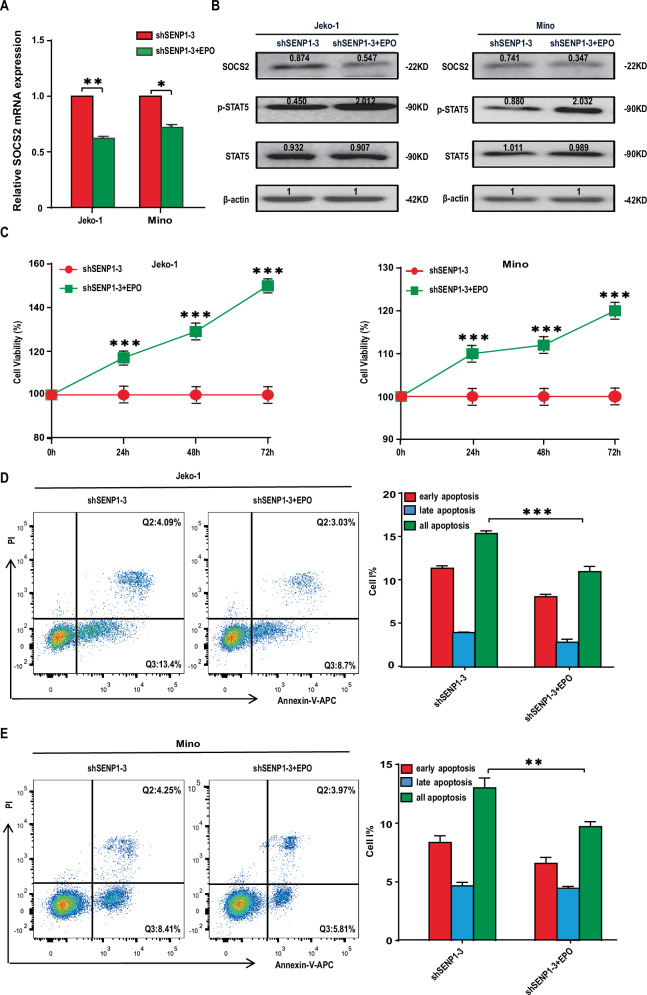
Erythropoietin (EPO) rescues the effect of SENP1 knockdown on MCL cells. SOCS2 mRNA levels were detected by RT-qPCR (**A**), and the protein levels of SOCS2, p-STAT5 and STAT5 were detected by western blot (**B**) in shSENP1-3 transfected Jeko-1 and Mino cells after treated with EPO for 24 h. **C** The proliferation of shSENP1-3 transfected Jeko-1 and Mino cells was determined by CCK8 array after treated with EPO for 0 h, 24 h, 48 h or 72 h. The apoptosis of shSENP1-3 transfected Jeko-1 (**D**) and Mino (**E**) cells was measured by Annexin V-APC and PI double staining and flow cytometric analysis after treated with EPO for 48 h. Representative flow cytometry patterns from different groups were shown. Data were presented as mean ± SD and analyzed by two-sided Student’s *t* test. *n* = 3; **P < 0.01, and ***P < 0.001.

To investigate the mechanism by which SENP1 exerted its effects on MCL cells, we performed mRNA-seq of stable Jeko-1 cells with shSENP1-3 or with shCon. As shown in Fig. 4A, multiple differentially expressed genes (DEGs) were found to be significantly differentially expressed genes between shSENP1 group and shCon group. KEGG enrichment analysis showed that the statistically significant DEGs were involved in various signaling pathways, including JAK-STAT pathway (Fig. 4B). GSEA further revealed that SENP1 expression was significantly associated with the JAK-STAT pathway (Fig. 4C). Among the significant DEGs, SOCS2 has been reported as a tumor suppressor and its expression is regulated by the JAK-STAT5 pathway. We found SOCS2 mRNA expression was significantly downregulated in MCL specimen compared with normal specimens by extracting data from GEO database (Fig. 4D). Pearson correlation analysis between SENP1 mRNA level and SOCS2 mRNA level in the same samples showed that there was a significantly negative correlation (*r* = −0.373, *P* = 0.035; Fig. 4E) between the two molecules. In Jeko-1 and Mino cells, both the mRNA and protein levels of SOCS2 were significantly upregulated by SENP1 knockdown (Fig. 4F, G). Moreover, knockdown of SENP1 in Jeko-1 and Mino cells deregulated p-STAT5 expression without significant influence of STAT5 expression (Fig. 4H). As previous studies demonstrated that EPO could activate the phosphorylation of Tyr694 of STAT5 (ref. [19, 20]), we treated Jeko-1-shSENP1 and Mino-shSENP1 cells with EPO, and tested the level of phosphorylation of STAT5, we found that EPO can activate the phosphorylation of STAT5 in Jeko-1 and Mino cells and inhibit expression of SOCS2 (Fig. 5A, B). We also tested proliferation and apoptosis of these cell lines, and found EPO could rescue the effect of SENP1 knockdown on MCL cell proliferation and apoptosis, at least partially (Fig. 5C–E). Together, the results suggest that SENP1 knockdown promotes SOCS2 expression through inhibiting JAK-STAT5 pathway.

### SENP1 knockdown suppresses MCL growth in vivo

**Fig. 6:**
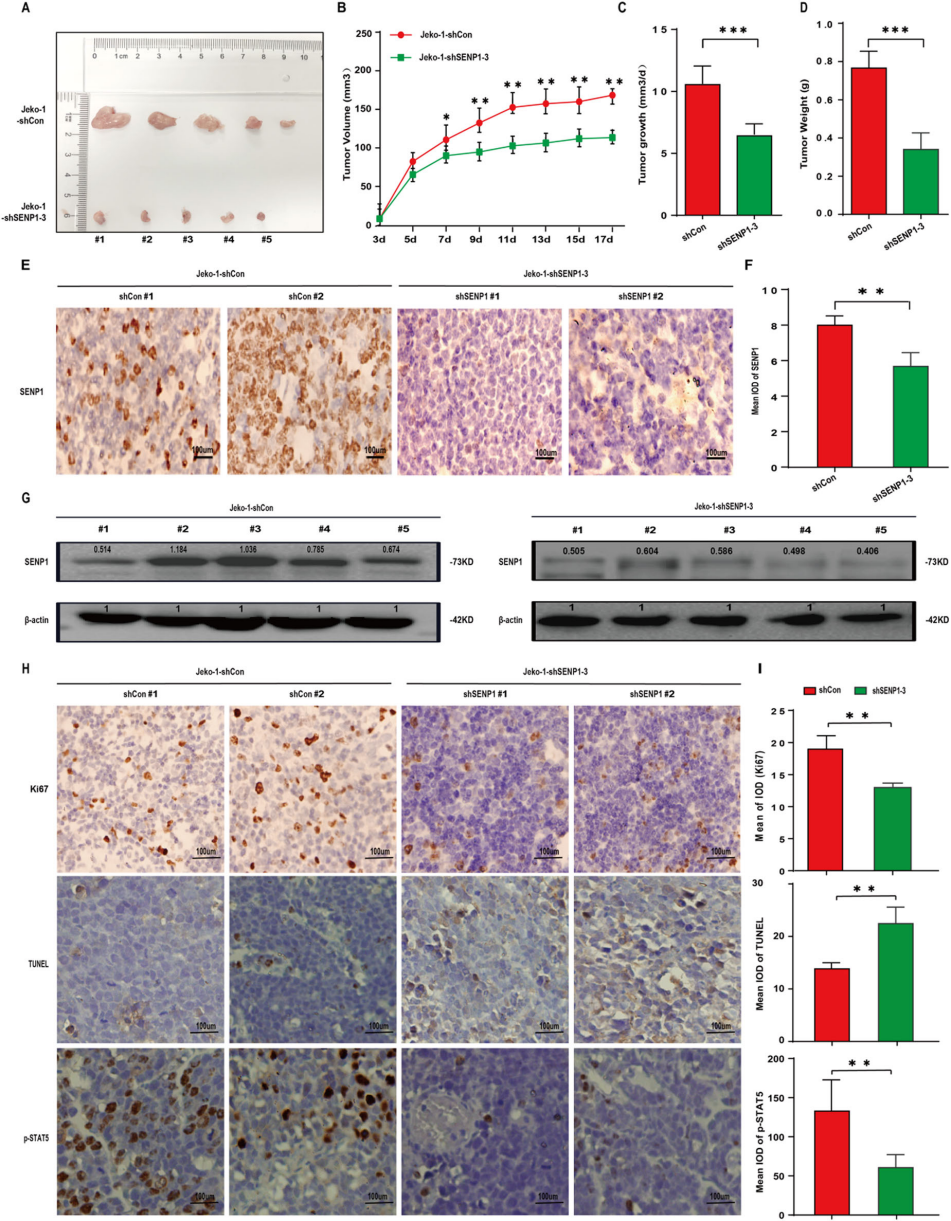
SENP1 knockdown suppresses MCL growth in vivo. **A** Images of the MCL xenografts in each mouse subcutaneously injected with shCon or shSENP1-3 transfected Jeko-1 cells (*n* = 5 per group). **B** Tumor volume in shCon and shSENP1-3 groups was recorded every two days for total 17 days. **C** Tumor growth rate was compared between shCon and shSENP1-3 groups. **D** Tumor weight was compared between shCon and shSENP1 groups. **E** Representative immunohistochemical images of SENP1 staining in MCL xenografts from shCon and shSENP1-3 groups. **F** The mean integrated optical density (IOD) of SENP1 protein in MCL xenografts from shCon and shSENP1-3 groups was calculated by Image-Pro Plus 6.0 software. **G** SENP1 protein level in each MCL xenograft from shCon and shSENP1-3 groups was measured by western blot. **H** Representative images of Ki67 staining, TUNEL assay and p-STAT5 in MCL xenografts from shCon and shSENP1-3 groups. **I** The mean integrated optical density (IOD) of Ki67, TUNEL and p-STAT5 in MCL xenografts from shCon and shSENP1-3 groups were calculated by Image-Pro Plus 6.0 software. Data were presented as mean ± SD and analyzed by two-sided Student’s *t* test. *n* = 3; *P < 0.05, **P < 0.01, and ***P < 0.001.

The in vivo effect of SENP1 on MCL growth was further evaluated in a xenograft nude mouse model. Stable Jeko-1 cells with shCon or shSENP1-3 were injected to nude mice to form subcutaneous tumors. The tumors formed by SENP1 knockdown Jeko-1 cells grew significantly slower than the tumors formed by Jeko-1 cells with shCon (Fig. 6A, B). Tumor growth rate was calculated by the equation of [tumor volume at day 21 - tumor volume at day 0]/21, and it was lower in the SENP1 knockdown group than in the control group (Fig. 6C). At day 21, tumors were isolated from the two group mice and then weighted. The tumors in the SENP1 knockdown group were significantly lighter (Fig. 6D). A significantly deregulated SENP1 expression was demonstrated in tumor specimens of the SENP1 knockdown group compared with control tumor specimens (Fig. 6E–G). Cell proliferation was significantly inhibited, cell apoptosis was significantly increased, and p-STAT5 protein expression was significantly down-regulated in the SENP1 knockdown group compared with the control group (Fig. 6H, I), which were consistent with the results in vitro.

## Discussion

Mantle cell lymphoma (MCL) is an aggressive B-cell lymphoma which usually presents at a late stage and is associated with a poor prognosis [21]. While high response rates are observed with induction chemo-immunotherapy, relapse is unfortunately universal in daily clinical practice. So deep understanding of the pathogenesis of this disease will be helpful for designing proper targeted therapeutic regimen and thereafter improving the prognosis.

The results presented here provide several lines of evidence indicating that SENP1 is involved in the pathogenesis of MCL. First, we found that SENP1 was highly expressed in MCL through immunohistochemical staining of MCL samples and analysis of GEO database. Then, we found that knockdown of SENP1 can inhibit the proliferation and promote the apoptosis of MCL cell lines. We also found that SENP1 could regulate the activity of JAK-STAT5 pathway and then affect the expression of SOCS2. Finally, we demonstrated these findings in mouse xenograft model.

Protein function is tightly regulated by reversible posttranslational modifications to create an on/off state that is crucial for many biological processes. Many proteins are dynamically modified at multiple sites by different modifications. SUMOylation is a new protein post-translational modification discovered in recent years [22–24], which can directly regulate a number of cellular processes, including transcription, DNA repair, cell cycle, and signal transduction. The balance between SUMOylation and deSUMOylation is regulated by SENPs [25, 26]. The abnormality of SENPs is involved in the occurrence and progression of many tumors [10, 27, 28]. SENP1 is the most potent protease for regulating SUMOylation, which is a hot molecule in the studies on post-translational modifications. The abnormality of SENP1 has been proved to be closely related to the occurrence and development of a variety of tumors. On the basis of other researchers, our results demonstrated the role and potential mechanism of SENP1 in pathogenesis of MCL for the first time.

In our study, we demonstrated that SENP1 could regulate the phosphorylation of STAT5 by biochemical study in MCL cell lines. The interplay between phosphorylation and SUMOylation of neighboring sites has been shown to play an important role in regulating the transcriptional activity of several transcription factors [29, 30]. Previous reports also demonstrated that SENP1 can regulate the interplay between STAT5 SUMOylation and phosphorylation which participates in the early lymphoid development [11]. We postulated that in MCL, this might be also the molecular mechanism that SENP1 regulate phosphorylation of STAT5 since there are underling similarity between lymphoid progenitor cells and malignant lymphoma cells as reported in previous literatures.

Besides the potential direct regulation of SUMOylation and phosphorylation of STAT5, we also found that SENP1 could regulate the expression of SCOS2, and hypothesized an underlying signaling axis of SENP1/JAK-STAT5/SOCS2 in MCL cells. The molecular basis for the hypothesis of this signaling axis lies those previous studies have demonstrated that STAT5 could regulate the expression of SOCS2 in mRNA level and protein level. Sen et al. showed in their study that STAT5 inhibition could decrease SOCS2 expression in head and neck squamous carcinoma [31]. More important, Steven et al. directly demonstrated that STAT5 activation/inactivation could regulate expression of SOCS2 in lymphoma cells [32]. This mechanism was further verified by Hu et al. in dendritic cells. SOCS2 is usually being considered as a tumor suppressor in multiple cancers [33]. So, our results in combination with previous works demonstrated that highly expressed SENP1 could activate STAT5 through regulation the interplay between SUMOylation and phosphorylation in MCL cells, then, activated STAT5 could inhibit the expression of tumor suppressor SCOS2 (ref. [31, 33–36]). This signaling pathway could explain the mechanism of SENP1 in pathogenesis of MCL.

Taken together, we found SENP1 was upregulated in MCL patients and cell lines; knockdown of SENP1 inhibited MCL cell proliferation and promoted cell apoptosis, and also inhibited MCL tumor growth in mice; knockdown of SENP1 resulted in inhibition of JAK-STAT5 pathway and increased the expression of tumor suppressor SOCS2. These findings indicated SENP1 as a new player in the pathogenesis mechanism of MCL and also a potential therapeutic target for MCL patients. Specific inhibitors of SENP1 have also shown good in vitro therapeutic effects in a variety of tumors, which provides the possibility of clinical transformation for treatments targeting this molecule in MCL patients.

## Supplementary information

Supplemental material
