# Correction: SENP1 promotes MCL pathogenesis through regulating JAK-STAT5 pathway and SOCS2 expression

**DOI:** 10.1038/s41420-025-02347-6

**Published:** 2025-04-08

**Authors:** Yali Zhang, Yanni Ma, Guixian Wu, Mingling Xie, Chengxin Luo, Xiangtao Huang, Feng Tian, Jieping Chen, Xi Li

**Affiliations:** 1 Department of Hematology, Southwest Hospital, Army Medical University (Third Military Medical University), Chongqing, China; 2 Department of Hepatobiliary Surgery, Southwest Hospital, Army Medical University (Third Military Medical University), Chongqing, China; 3 Institute of Infectious Diseases, Southwest Hospital, Army Medical University (Third Military Medical University), Chongqing, China

Correction to: *Cell Death Discovery* 10.1038/s41420-021-00578-x, published online 26 July 2021

The original version of this article contained an error. In Fig. 6H, the upper-left picture and the upper-right picture were mistakenly uploaded during assembling images for the Fig. 6. The revised Fig. 6 that contains the correct pictures showing the results of the Ki67 staining from shCon#1 group and from shSENP1#2 group, as well as the corresponding bar graph (the upper picture in Fig. 6I), is shown below. This correction does not influence the conclusions of the article. The authors apologize for any confusion caused by this error.

Original Figure 6
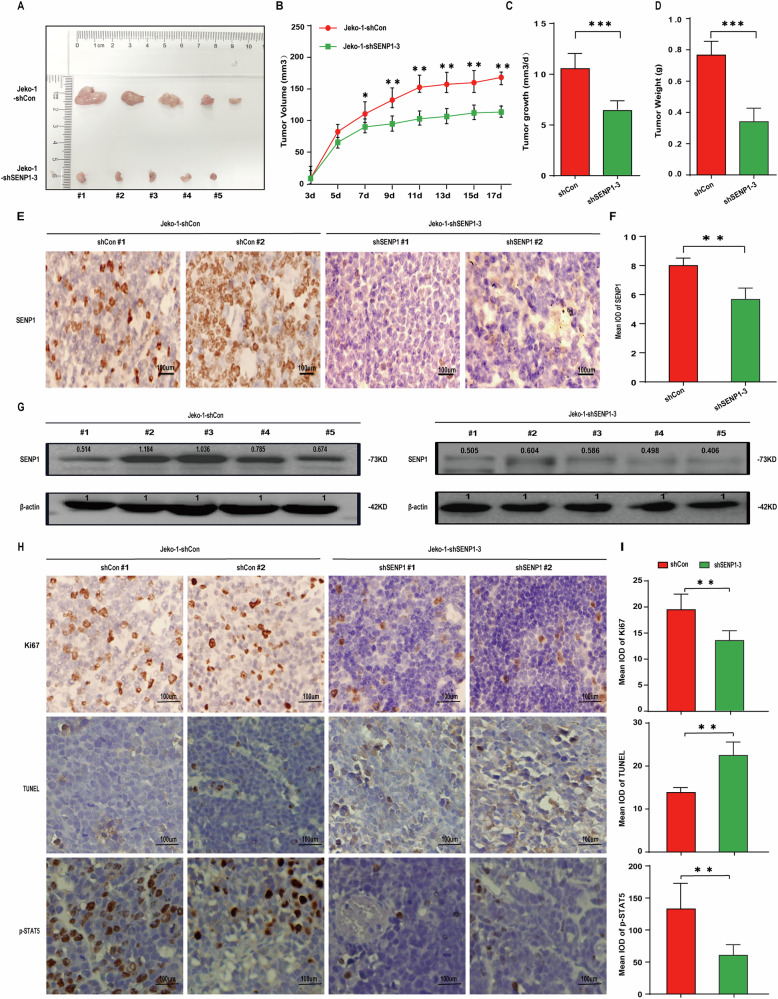


Corrected Figure 6
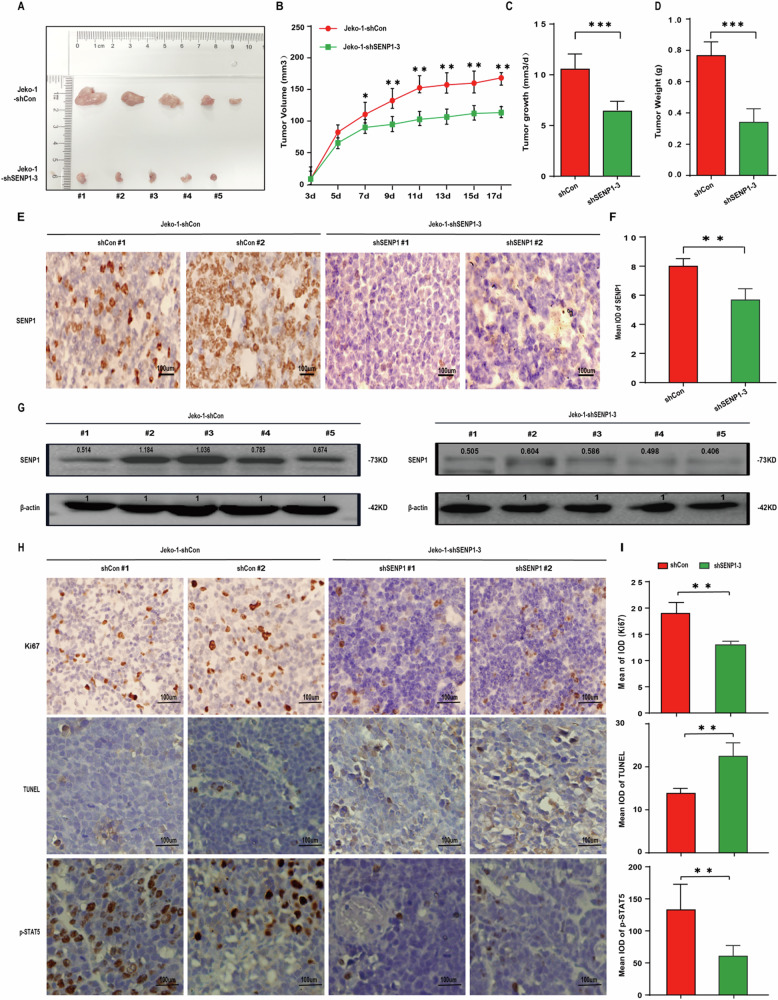


The original article has been corrected.

