# Supplementary material for: SENP1 promotes MCL pathogenesis through regulating JAK-STAT5 pathway and SOCS2 expression
Source: Cell Death Discov. 2021 Jul 26;7:192. doi: 10.1038/s41420-021-00578-x (PMC8313533; doi:10.1038/s41420-021-00578-x)
Supplement: Supplementary file 1 — Supplemental material [file 41420_2021_578_MOESM1_ESM.doc]

Table S1 Sequences utilized in this study.

| **Genes** | **Forward (5’-3’)** | **Reverse (5’-3’)** |
| --- | --- | --- |
| SENP1 | GGTGACCTTCGACCGCAGTGTT | TGGGTGATGCCGTTGATGTTGC |
| SOCS2 | TAACCCGGGACTTGGA GAAG | GCTTCTTGGGCGTCTGCTC |
| GAPDH | AGGCCGGTGCTGAGTATGTC | CGGTACAACGAGCTGTTTCTAC |
| shRNA sequences(5’-3’) | | |
| shSENP1-1 | TGACCATTACACGCAAAGATA | |
| shSENP1-2 | GCGCCAGATTGAAGAACAGAA | |
| shSENP1-3 | CTCGATGTCTTAGTTCCAGTA | |
